# Lysosomal acid lipase in cancer

**DOI:** 10.18632/oncoscience.223

**Published:** 2015-08-30

**Authors:** Cong Yan, Ting Zhao, Hong Du

**Affiliations:** Department of Pathology and Laboratory Medicine and IU Simon Cancer Center, Indiana University School of Medicine, Indianapolis, IN, USA

**Keywords:** MDSC, mTOR, PPARgamma, cancer, lysosomal acid lipase

Lysosomal Acid Lipase Regulates Myeloid-derived Suppressor Cells to Control Cancer Cell Proliferation and Metastasis. Inflammation critically contributes to cancer growth and metastasis, in which myeloid-derived suppressor cells (MDSCs) are an important participant. MDSCs are known to suppress immune surveillance to promote tumorigenesis [1]. Lysosomal acid lipase (LAL), a critical enzyme in controlling neutral lipid metabolic signaling, hydrolyzes cholesteryl esters and triglycerides in the lysosome of cells to generate free fatty acids and cholesterol. Deficiency of this metabolic enzyme causes abnormal hematopoietic development, skewing progenitor cell differentiation towards an overabundance of myeloid cells in the bone marrow. As a result, immature MDSCs expand dramatically and accumulate in the bone marrow, peripheral blood, immune organs (e.g. thymus, spleen) and distal organs (e.g. lung, liver) [2]. Systemic infiltration of *lal−/−* MDSCs is facilitated by changed ECs functions through various mechanisms. *lal−/−* MDSCs promote ECs angiogenesis, tube formation, and proliferation [3].

Upon exiting the bone marrow and entering circulation and distal organ compartments, *lal−/−* MDSCs promote tumorigenesis by two mechanisms. First, infiltration of *lal−/−* MDSCs leads to abnormal organization of the thymus and spleen, impair progression of T cell development in the thymus, and retard T cell maturation in the spleen. In the blood and distal organs, *lal−/−* MDSCs strongly suppress peripheral T cell proliferation and lymphokine release [4]. This is largely due to increased apoptosis and decreased proliferation of *lal−/−* T cells in thymus and peripheral compartments. As a consequence, the anti-tumor immunity is significantly weakened. Second, *lal−/−* MDSCs directly stimulate tumor cell proliferation, growth and metastasis even in an allogenic mouse model, showing that *lal−/−* MDSCs are able to overcome host immune rejection [5]. Therefore, MDSCs facilitate tumorigenesis at least by two mechanisms: 1) suppressing immune surveillance; and 2) stimulating cancer cell proliferation, growth and metastasis directly.

Based on Affymetrix GeneChip microarray and Ingenuity Pathway analyses, the mammalian target of rapamycin (mTOR) signaling and peroxisome proliferator-activated receptor gamma (PPARγ) signaling are two major pathways that mediate pathogenic phenotypes of *lal−/−* MDSCs in suppressing immune surveillance and stimulating tumorigenesis [6]. These activities are associated with alteration of other genes involved in cell growth, cell cycle entry, cell survival, cell migration, histone epigenetics, bioenergetic pathways, and ROS production [6]. Inhibition of mTOR by pharmacological inhibitors and siRNA interference not only reduces immunosuppressive function of *lal−/−* MDSCs, but also suppresses their stimulation on tumor proliferation, progression and metastasis [5, 7]. mTOR inhibitor treatment of *lal−/−* mice reverses the increased proliferation, decreased apoptosis, increased ATP synthesis, and increased cell cycling in *lal−/−* MDSCs. Pharmacological and siRNA suppression of mTOR, Raptor, Rictor, and Akt1 also correct *lal−/−* MDSCs development from Lin-progenitor cells, decrease ROS production, and recover from impairment of mitochondrial membrane potential.

On the other hand, derivatives of free fatty acid metabolites serve as hormonal ligands for nuclear receptors (e.g. PPARγ) that possess the anti-inflammatory function. Myeloid-specific expression of human LAL (hLAL) to restore the PPARγ function in *lal*−/− mice reverses both immunosuppression and tumor stimulation [2, 5]. Reintroducing PPARγ ligands into *lal*−/− mice also reverses immunosuppression and tumor stimulation (unpublished result). To confirm the functional role of PPARγ in myeloid cells, dominant negative PPARγ (dnPPARγ) was overexpressed in a myeloid-specific bitransgenic mouse model [8]. Overexpression of dnPPARγ in myeloid lineage cells abnormally elevates frequencies and total numbers of LK, LSK, CMP and GMP progenitor populations in the bone marrow. As a result, MDSCs are systemically increased in association with activation of Stat3, NF-kB, Erk1/2 and p38 molecules. MDSCs from this system inhibit the proliferation and lymphokine production of T cells. Both CD4+ and CD8+ T cell populations are decreased in conditional bitransgenic mice. Multiple forms of carcinoma and sarcoma in the lung, liver, spleen and lymph nodes are observed. Bone marrow transplantation reveals that a myeloid autonomous defect is responsible for MDSCs expansion, immunosuppression and tumorigenesis in this conditional bitransgenic model [8]. Therefore, the metabolic signaling controlled by the LAL/PPARγ/mTOR axis is essential for MDSCs development, homeostasis and functions during tumorigenesis, which can facilitates pharmacological drug and immunotherapy designs in clinical application.
